# Knockdown of Keratin 6 Within Arsenite-Transformed Human Urothelial Cells Decreases Basal/Squamous Expression, Inhibits Growth, and Increases Cisplatin Sensitivity

**DOI:** 10.3390/cells13211803

**Published:** 2024-10-31

**Authors:** Nelofar Nargis, Donald A. Sens, Aaron A. Mehus

**Affiliations:** Department of Pathology, School of Medicine and Health Sciences, University of North Dakota, Grand Forks, ND 58202, USA; nelofar.nargis@und.edu (N.N.); donald.sens@med.und.edu (D.A.S.)

**Keywords:** keratin 6, basal, urothelial carcinoma, arsenite, cisplatin, squamous differentiation

## Abstract

Urothelial carcinoma (UC) is prevalent, especially in elderly males. The high rate of recurrence, treatment regime, and follow-up monitoring make UC a global health and economic burden. Arsenic is a ubiquitous toxicant that can be found in drinking water, and it is known that exposure to arsenic is associated with UC development. Around 25% of diagnosed UC cases are muscle-invasive (MIUC) which have poor prognosis and develop chemoresistance, especially if tumors are associated with squamous differentiation (SD). The immortalized UROtsa cell line is derived from normal human urothelium and our lab has malignantly transformed these cells using arsenite (As^3+^). These cells represent a basal subtype model of MIUC and the tumors derived from the As^3+^-transformed cells histologically and molecularly resemble clinical cases of the basal subtype of MIUC that have focal areas SD and expression of the basal keratins (KRT1, 5, 6, 14, and 16). Our previous data demonstrate that KRT6 protein expression correlates to areas of SD within the tumors. For this study, we performed a lentiviral knockdown of KRT6 in As^3+^-transformed UROtsa cells to evaluate the effects on morphology, gene/protein expression, growth, colony formation, and cisplatin sensitivity. The knockdown of KRT6 resulted in decreased expression of the basal keratins, decreased growth, decreased colony formation, and increased sensitivity to cisplatin, the standard treatment for MIUC. The results of this study suggest that KRT6 plays a role in UC cell growth and is an exploitable target to increase cisplatin sensitivity for MIUC tumors that may have developed resistance to cisplatin treatment.

## 1. Introduction

Muscle-invasive urothelial cancer (MIUC) presents or develops in approximately 25% of patients diagnosed with bladder cancer [1,2]. The MIUCs are characterized as more aggressive, chemotherapy-resistant, and having a poor outcome with a very low 5-year survival rate [3]. There has been a concerted effort to employ global gene expression profiling to define the subtypes of bladder cancer and their prognostic significance for patient outcomes. These studies followed the strategy previously used for patients with breast cancer, which identified molecular subtypes (basal/triple-negative, HER2+, luminal A, and luminal B) that behave clinically as distinct disease entities [4]. These efforts in bladder cancer have defined the gene expression signatures of bladder cancer and proposed several intrinsic subtypes of the disease [5,6,7,8,9]. A general finding in the above studies was the association of more basal subtypes with aggressive disease, including the enrichment of genes and histology features associated with squamous differentiation (SD). This was effectively illustrated for MIUCs, where basal tumors show more aggressive disease at presentation and are significantly enriched for squamous features [6,10] Overall, SD is present in 20% of bladder cancers and is associated with a poor prognosis regardless of initial grade [11]. Squamous differentiation is focally present within the tumor and the degree of SD may have some influence on prognosis [11,12,13].

For the past four decades, cisplatin-based treatment has been the gold standard for treating MIUC [14]. This cisplatin-based treatment is typically given as neoadjuvant chemotherapy (NAC) prior to radical cystectomy. However, patients frequently acquire cisplatin resistance. Recent studies have described alternative treatment options that include different chemotherapies or combinations with cisplatin to increase efficacy in combating advanced UC [15,16].

This laboratory has shown that keratin 6 (KRT6) immunostaining could identify areas of SD in human UC, even in very small areas of focal SD [17]. The significance of this finding was increased when SD and its associated genes, including KRT6, were found in the above studies to be a marker of poor prognosis for patients with UC. Our laboratory also developed a cell culture and transplant model of human UC employing the immortalized, but not tumorigenic, UROtsa cell line through exposure to arsenite (As^3+^) or cadmium (Cd^2+^) [18,19]. The tumor transplants generated from the cultured cells showed a transitional cell morphology with focal areas of SD that could be identified by KRT6 immunostaining [17,20]. Subsequent studies showed that the transplanted tumors produced by the transformed UROtsa cells expressed the basal subtype of human MIUC, including KRT6 [21]. An examination of the urospheres isolated from the parent and As^3+^-transformed cells also showed an identity with the basal subtype of human MIUC, including KRT6 expression [22].

The expression of KRT6 in normal tissues has been reported in basal cells of respiratory epithelium and in the suprabasal compartment of non-keratinizing stratified squamous epithelia [23]. A previous study from this laboratory showed that the KRT6 protein was not expressed in normal urothelium [17]. KRT6 has been associated with various hyperproliferative epidermal disorders, suggesting these keratins may be molecular markers for hyperproliferative keratinocytes [23,24,25]. They are not expressed in non-proliferating skin keratinocytes, but during skin wounding KRT6 is rapidly induced at the wound edge before migration and regeneration begins to occur [25,26,27]. Once healing is complete, the expression of KRT6 is downregulated to undetectable levels. Null mouse models have established the role of KRT6 in maintaining cell adhesion and optimal cell migration. Germline deletion of KRT6 results in the birth of normal-appearing mice, but death ensues in the first week after birth due to oral epithelial blistering and lack of nutrition [28]. There have been limited studies, if any, on the knockdown of KRT6 in vitro to determine if it functions solely as a terminal keratin in cell differentiation or if it can influence the expression of other regulatory aspects of growth or gene expression.

The goal of the present study was to knock down KRT6 expression in As^3+^-transformed UC cells in vitro to determine if growth or key genes/proteins implicated in UC with SD are altered through the inhibition of KRT6 expression. The basal keratins (KRT1, KRT5, KRT14, and KRT16) and additional genes involved with SD such as: the small proline-rich family of proteins (*SPRR1A*, *SPRR2A*, *SPRR3*) and desmocollin 2 (*DSC2*), were chosen for the initial analysis. We then assessed cellular growth rates and colony formation abilities after the stable knockdown of KRT6, and analyzed the expression levels of epidermal growth factor receptor (EGFR) and aldehyde dehydrogenase 3 family member A1 (ALDH3A1), two proteins involved with the maintenance of cancer stem cells [29,30].

## 2. Materials and Methods

### 2.1. Cell Culture

Two separate UROtsa As^3+^-transformed isolates (As_I and As_II) were cultured at 37 °C and 5% CO_2_ in Dulbeco’s modified Eagle’s medium (DMEM) supplemented with 5% *v*/*v* fetal bovine serum (FBS), as described previously [19]. The cells were sub-cultured at a 1:10 split ratio using TrypLE (Gibco, Waltham, MA, USA) and the cultures were fed fresh growth medium every three days. The UROtsa parent cell line was authenticated using short tandem repeat (STR) analysis [31]. The As^3+^-transformed isolates used in the current study were previously characterized for their ability to form colonies in soft agar, form spheroids when grown in ultra-low attachment flasks and to form tumors when injected subcutaneously in immune-compromised mice [19,20,31,32,33]. 

### 2.2. GEPIA Gene Expression and Gene Correlations

The transcript expression boxplots for *KRT1*, *KRT5*, *KRT6A*, *KRT14*, and *KRT16* in normal vs. human bladder urothelial carcinoma (BLCA) was obtained using Gene Expression Profiling Interactive Analysis 2 (GEPIA2, http://gepia2.cancer-pku.cn/#index accessed on 25 September 2024) [34]. The *p*-value cutoff was 0.01 (*p* < 0.01). The correlation analysis was performed using a BLCA tumor with the Pearson correlation coefficient. A correlation between gene expression was considered significant when *p* < 0.05.

### 2.3. Lentivirus Transduction

The lentiviral particles used in this study (control and KRT6 shRNA) were sequence A: pLV[Exp]-U6 > {hKRT6A,B,C[shRNA]}: Terminator-CMV > Luciferase(ns): P2A:Puro (Vector ID: VB231219-1383ftk), sequence B: pLV[Exp]-U6 > hKRT6A,B,C[shRNA#2]: Terminator-CMV > Luciferase(ns): P2A:Puro (Vector ID: VB231221-1894cvb), and scramble control pLV[Exp]-U6 > Scramble[shRNA#1]: Terminator-CMV > Luciferase(ns): P2A:Puro (Vector ID: VB231221-1036rcq). The lentiviral particles were constructed and packaged by VectorBuilder. The vector ID can be used to retrieve detailed information about the vector on www.vectorbuilder.com. The UROtsa As_I and As_II cells were seeded in a 12-well plate (100,000 cells/well) and infected overnight in medium containing lentiviral particles and polybrene (5 µg/mL). The following morning, the old media was replaced with fresh media for 24 h before selection with puromycin (2 µg/mL). Puromycin selection lasted for seven days during which the cells were fed fresh puromycin containing media every three days. 

### 2.4. Colony Formation Assay

UROtsa As_I and As_II cells were grown in 6-well plates (200 cells/well). There were triplicate seedings on the 6-well plate of the control and KRT6 knockdown cells for each cell line (As_I and As_II). The cells were cultured for 14 days, with the medium changed every three days. The resulting colonies were stained with 1 mL of 0.5% crystal violet (Sigma-Aldrich Corp., St. Louis, MO, USA) in 20% methanol for 15 min at room temperature. The plate was washed five times in fresh water, allowed to dry, and colonies counted using ImageJ (v1.53) software. 

### 2.5. Tag-It Violet Proliferation Assay

The Tag-it violet proliferation assay has been described previously [35]. Briefly, six million cells were incubated with 1 mL Phosphate-Buffered Saline (PBS) that contained 5 µM Tag-IT violet dye (BioLegend, San Diego, CA, USA, #425101) for 30 min at 37 °C, after which five milliliters of DMEM medium with 5% FBS was added to terminate the reaction, and the cells were centrifuged at 2000 rpm for 5 min. Cells were resuspended in fresh DMEM medium with 5% FBS and seeded into six-well plates at a density of 250,000 cells/well and were cultured for seventy-two hours. The cells were trypsinized and collected in Falcon tubes (Corning, #352058, Glendale, AZ, USA) in 500 µL fluorescence-activated cell sorting (FACS) buffer (PBS + 5% FBS). Samples were acquired using a Sony (SH800, San Jose, CA, USA) flow cytometer and analyzed with FlowJo software (v10.10.0). 

### 2.6. RNA Isolation and Real-Time PCR Analysis 

The following RNA isolation and real-time PCR analysis has previously been described [35]. The cells were washed twice with PBS then total RNA was isolated from cells using the RNeasy Plus Mini Kit, Qiashredders, and QiaCube instrument following the manufacturer’s protocols (Qiagen, Hilden, Germany). Total RNA (1 µg) was transcribed into cDNA using LunaScript^®^ RT SuperMix Kit (New England Biolabs #E3010L, Ipswich, MA, USA) according to the manufacturer’s protocol. cDNA (2 µL) was combined with 0.2 µM primers and Luna^®^ Universal qPCR Master Mix (New England Biolabs # M3003E) according to the manufacturer’s protocol in a total volume of 20 µL. qPCR gene amplification was monitored via SYBR Green fluorescence using BioRad CFX96 Touch Real-Time PCR Detection System. qPCR cycle conditions were 1 cycle of 2 min at 95 °C, and 40 cycles of 5 s at 95 °C and 30 s at the annealing temperature of 60 °C. Expression levels were determined from the threshold cycle (Ct) values using the method of 2^−∆∆Ct^ using 18S as the reference control gene. Primers were obtained from Integrated DNA Technology (IDT, Coralville, IA, USA) and are listed in Table S1. 

### 2.7. Simple Western Blot Analysis

The use of simple Western blot to measure protein expression has been previously described [35,36,37,38,39]. Briefly, the cells were washed twice with PBS then dissolved in 1X Radio-immunoassay Precipitation Assay (RIPA) lysis buffer supplemented with PMSF, protease inhibitor cocktail, and sodium orthovandate (Santa Cruz Biotechnology, Dallas, TX, USA). The cell suspension was sonicated and the lysate was centrifuged to remove cellular debris. Protein lysates were quantified using the Pierce BCA protein assay kit (Thermo-Scientific Pierce). Diluted protein lysates were combined with 5X fluorescent master mix (ProteinSimple, San Jose, CA, USA), which contains a reducing agent (dithiothreitol), fluorescent standards, and a system control protein (26 kDa). Denatured (95 °C for 5 min) lysates were separated and the immunodetection of target proteins was performed using a capillary-based Jess Simple Western instrument (ProteinSimple, San Jose, CA, USA) according to the manufacturer’s protocol. The ProteinSimple system control protein (26 kDa) served as an internal control and was also used to normalize protein expression. Duplicates of each sample (4 µL) were analyzed for target protein expression. The protein lysate concentrations used to detect each target protein and antibodies/dilutions are listed in Table S2. The uncropped blots are provided in Figure S1.

### 2.8. Luciferase Assay for Growth/Viability

The control and KRT6 knockdown cells were seeded at various densities in a 96-well plate (156–5000 cells/well). For growth, the cells were seeded at 1250 cells/well. The cells were then cultured for 24 h and luminescence was read on a Tecan Spark plate reader 10 min after the addition of sterile filtered luciferin (Revvity Health Sciences Inc (Waltham, MA, USA, Cat. # 122799), 150 µg/mL). After luminescence was read, the luciferin-containing media was replaced with fresh media (no luciferin) and the cells were placed back in the incubator. Luminescence readings were obtained 24 h, 48 h, 72 h, and 144 h after seeding. For cell viability after cisplatin treatments, cells were seeded at 1250 cells/well. The cells were then cultured for 24 h and then treated with various doses of cisplatin-containing media. The treatment lasted 72 h before luminescence was measured. There were 8 replicates for each dose.

### 2.9. Statistical Analysis

Unless otherwise stated, experiments were performed in either duplicate or triplicate and the results are expressed as the mean ± SEM. Statistical analyses were performed using GraphPad Prism^®^ software version 10.3.1 using *t*-test. The level of significance was *p* < 0.05.

## 3. Results

### 3.1. Expression of Basal Keratins in Human Urothelial Carcinoma Tumors vs. Normal Tissue

**Figure 1:**
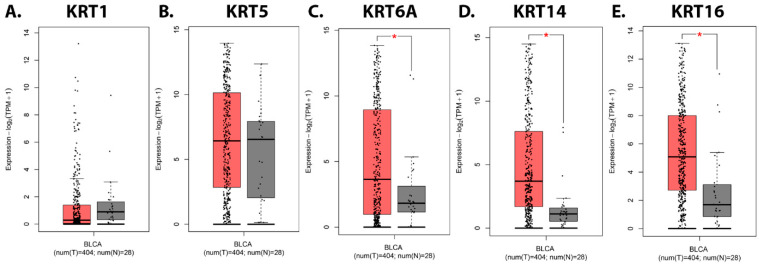
The expression of basal keratins in human urothelial carcinoma tumors vs. normal tissue. The GEPIA2 boxplot of (**A**) *KRT1*, (**B**) *KRT5*, (**C**) *KRT6A*, (**D**) *KRT14*, and (**E**) *KRT16* transcript expressions in normal and BLCA tissues. Red bars represent tumor (T) levels and grey bars represent normal (N) tissue levels. GEPIA, Gene Expression Profiling Interactive Analysis; BLCA, bladder urothelial carcinoma.

We first examined the transcript expression of keratins associated with the basal subtype of MIUC (*KRT1*, *KRT5*, *KRT6A*, *KRT14*, and *KRT16*) in human bladder urothelial carcinoma (BLCA) tumors vs. normal tissue using GEPIA2 (Gene Expression Profiling Interactive Analysis, http://gepia2.cancer-pku.cn/#index, accessed on 25 September 2024) [34]. Figure 1C–E demonstrates that *KRT6A*, *KRT14*, and *KRT16* transcript expression is higher in bladder tumor tissue than in normal tissue (*p* < 0.01). 

### 3.2. Knockdown of KRT6 and Morphology in As^3+^-Transformed UROtsa Cells

**Figure 2:**
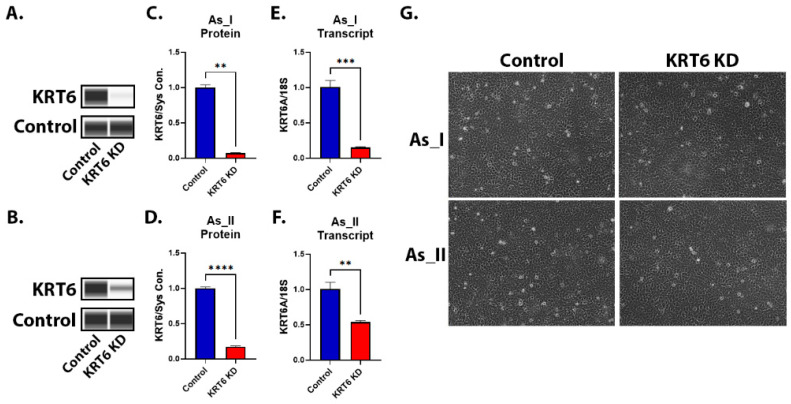
Knockdown of KRT6 and morphology in As^3+^-transformed UROtsa cells. (**A**,**B**) Western blot image of KRT6 expression after KRT6 knockdown in UROtsa As_I and As_II cells, respectively. (**C**,**D**) Quantification of KRT6 protein expression levels. (**E**,**F**) Quantification of mRNA transcript levels of *KRT6A*. (**G**) Brightfield microscope images (10× magnification) of UROtsa As_I and As_II cells after KRT6 knockdown. All data are plotted as fold-change compared to the scramble control for each cell line. Gene expression was normalized to the 18S housekeeping gene and protein expression was normalized to the Jess system control. The measurements were performed in triplicate for gene data and duplicate for protein data. The reported values are mean ± SEM. A *t*-test was performed, and asterisks indicate significant differences from the control (** *p* < 0.01, *** *p* < 0.001, **** *p* < 0.0001).

To efficiently knockdown the expression of KRT6 within our UROtsa As^3+^-transformed cells, we obtained two separate shRNA sequences that target human KRT6 and tested their ability to reduce KRT6 expression using Western blot in UROtsa As_I cells. Figure S2A,B shows that sequence “A” was the most effective (~90% reduction) in reducing KRT6 expression compared to sequence “B” or the combination of sequence “A” with sequence “B” (~60% reduction). Sequence “A” was used for the remainder of experiments in this manuscript. Figure 2A–F shows that KRT6 expression was reduced at the protein and transcript level in two separate As^3+^-transformed UROtsa cell lines (As_I and As_II). Figure S1 displays all the uncropped Western blot images used throughout the manuscript. The morphology of the cells was unchanged from knockdown of KRT6 (Figure 2G).

### 3.3. Expression of Basal Keratins After KRT6 Knockdown in As^3+^-Transformed UROtsa Cells

**Figure 3:**
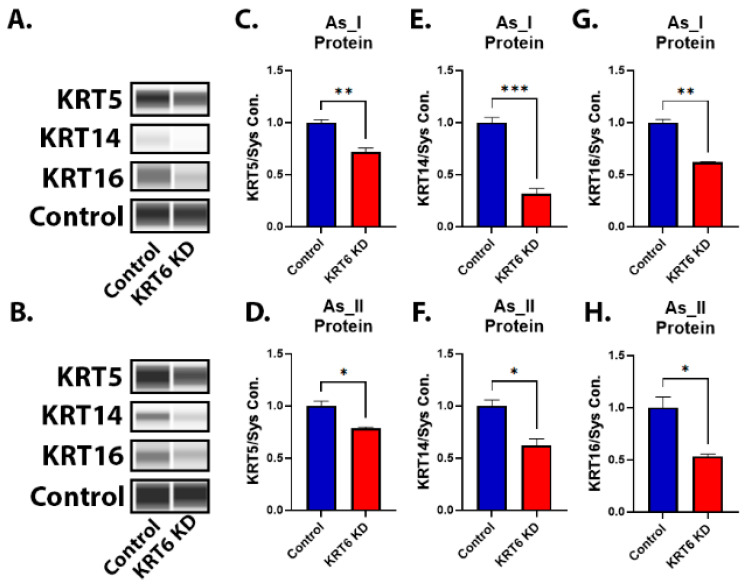
Expression of basal keratins after KRT6 knockdown in As^3+^-transformed UROtsa cells. (**A**,**B**) Western blot image of KRT5, KRT14, and KRT16 expression after KRT6 knockdown in UROtsa As_I and As_II cells, respectively. (**C**,**D**) Quantification of KRT5 protein expression levels. (**E**,**F**) Quantification of KRT14 protein expression levels. (**G**,**H**) Quantification of KRT16 protein expression levels. All data are plotted as fold-change compared to the scramble control for each cell line. Protein expression was normalized to the Jess system control. The measurements were performed in duplicate for protein data. The reported values are mean ± SEM. A *t*-test was performed and asterisks indicate significant differences from the control (* *p* < 0.05, ** *p* < 0.01, *** *p* < 0.001).

Since KRT6 is associated with the basal subtype of MIUC, we wanted to assess the expression of the other basal keratins (KRT1, KRT5, KRT14, and KRT16) after the knockdown of KRT6 in As_I and As_II cells. Figure 3A–H demonstrates that KRT5, KRT14, and KRT16 protein expression were also reduced in the UROtsa As_I and As_II cells after KRT6 knockdown. Figure S3A,B shows that the transcript of *KRT1* was reduced in both the cell lines after KRT6 knockdown. We also wanted to evaluate the correlation of expression between *KRT6A* and the other basal keratins in human bladder cancer using GEPIA2. The analysis demonstrated that human *KRT6A* expression is correlated (*p* < 0.05) to the expression of these other basal keratins in human bladder cancer cases (Figure S4A–D).

### 3.4. Expression of Genes Associated with SD After KRT6 Knockdown in As^3+^-Transformed UROtsa Cells

**Figure 4:**
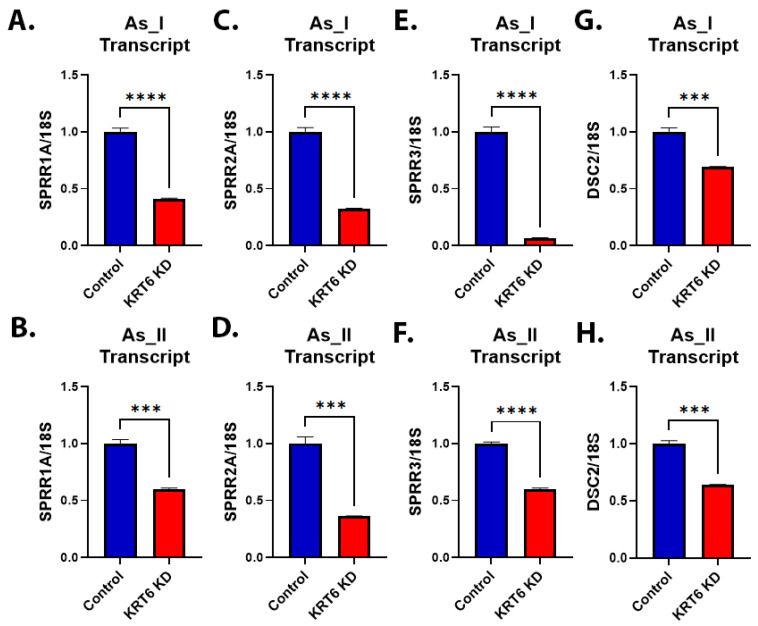
Expression of genes associated with SD after KRT6 knockdown in As^3+^-transformed UROtsa cells. (**A**,**B**) Quantification of mRNA transcript levels of SPRR1A after KRT6 knockdown in UROtsa As_I and As_II cells, respectively. (**C**,**D**) Quantification of mRNA transcript levels of SPRR2A. (**E**,**F**) Quantification of mRNA transcript levels of SPRR3. (**G**,**H**) Quantification of mRNA transcript levels of DSC2. All data are plotted as fold-change compared to the scramble control for each cell line. Gene expression was normalized to the 18S housekeeping gene. The measurements were performed in triplicate for gene expression data. The reported values are mean ± SEM. A *t*-test was performed, and asterisks indicate significant differences from the control (*** *p* < 0.001, **** *p* < 0.0001).

Small proline-rich family of proteins (SPRRs) and desmocollin 2 (DSC2) are enriched in areas of SD in UCs [40,41]. Furthermore, Figure S4E–H demonstrates that the expression of these squamous-associated genes is correlated to *KRT6A* expression in human UC cases. We next evaluated the expression of *SPRR1A*, *SPRR2A*, *SPRR3*, and *DSC2* in the UROtsa As_I and As_II cells after the knockdown of KRT6. Figure 4A–H demonstrates that the transcript level of all these squamous-associated genes was reduced after KRT6 knockdown in both cell lines. 

### 3.5. Signal Stability, Sensitivity, and Dynamic Range of Luminescence Assay for Growth of As^3+^-Transformed UROtsa Cells After KRT6 Knockdown

**Figure 5:**
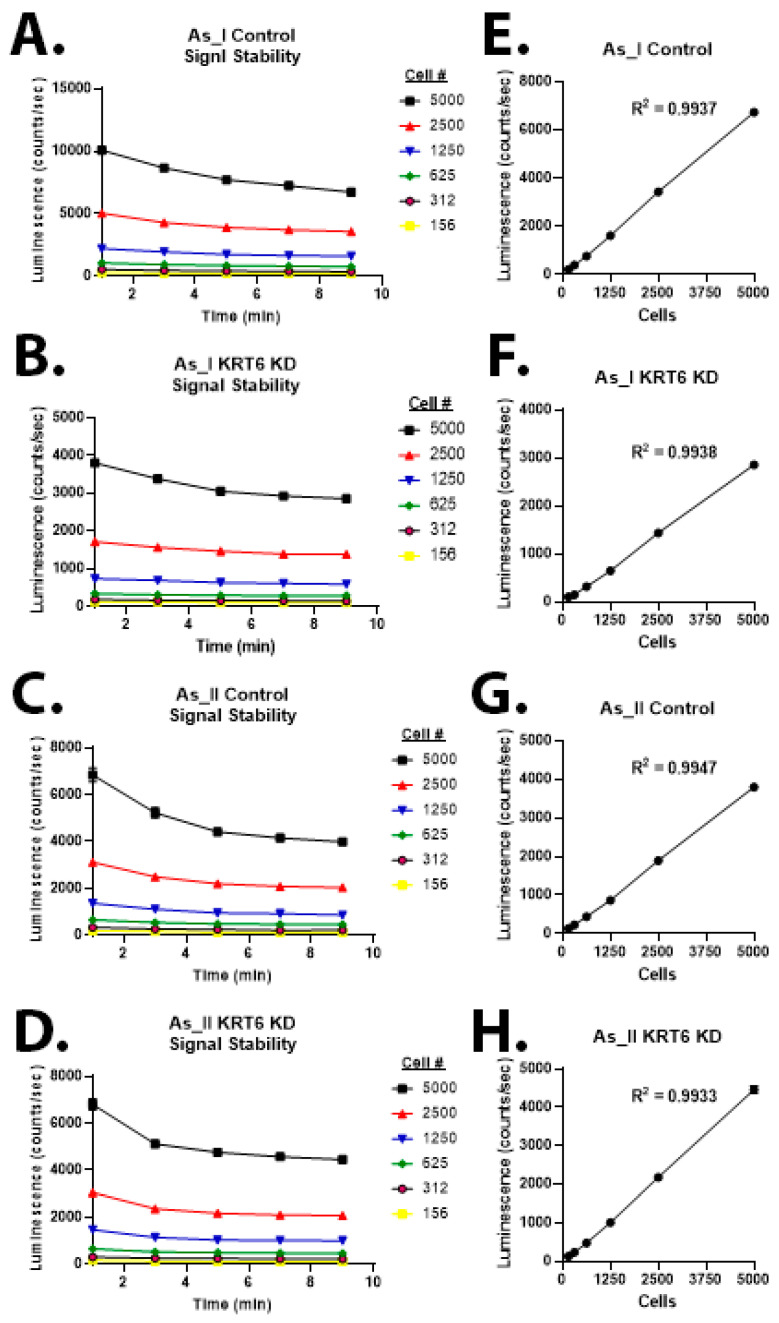
Signal stability, sensitivity, and dynamic range of luminescence assay for growth of As^3+^-transformed UROtsa cells after KRT6 knockdown. (**A**–**D**) Luminescence intensity over time showing that signal is stabilized by 10 min. (**E**–**H**) Luminescence intensity of two-fold serially diluted control and KRT6 knockdown UROtsa As_I and As_II cells. Dilution range was from 5000 to 156 cells/well. R^2^ shows the linear correlation between luminescent intensity and cell number. There were 8 replicates for each dilution on a 96-well plate and the mean intensity of the replicates was plotted.

We designed our lentiviral vectors to express a puromycin gene (for selection), our targeted KRT6 shRNA or scrambled control, and a luciferase reporter. Since our control and KRT6 knockdown cells express luciferase, we wanted to utilize this characteristic to develop a growth/viability assay to monitor the proliferation rate and drug sensitivity for these cells for future experiments. We first assessed signal stability (Figure 5A–D) in each of the control and KRT6 knockdown cells. The results demonstrate that the luminescence signal was stabilized 10 min after adding the substrate (luciferin diluted to 150 µg/mL). This is the timepoint that was chosen for all luminescence readings in the remaining experiments. After luminescence is read, the luciferin-containing media can be removed and replaced with fresh media. The whole process (incubation, reading, the addition of fresh media) is less than 15 min. This allows us to measure cellular growth from day-to-day luminescence readings using the same cells on the same plate to limit the technical variability that can be associated with multi-plate growth assays. We next wanted to test the sensitivity and dynamic range of the assay (Figure 5E–H). We performed a two-fold serial dilution starting from 5000 and reducing to 156 cells in a 96-well plate. The results demonstrate a good correlation (R^2^ < 0.99) between the luminescence intensity and the number of cells seeded for each cell line.

### 3.6. Growth of As^3+^-Transformed UROtsa Cells After KRT6 Knockdown

**Figure 6:**
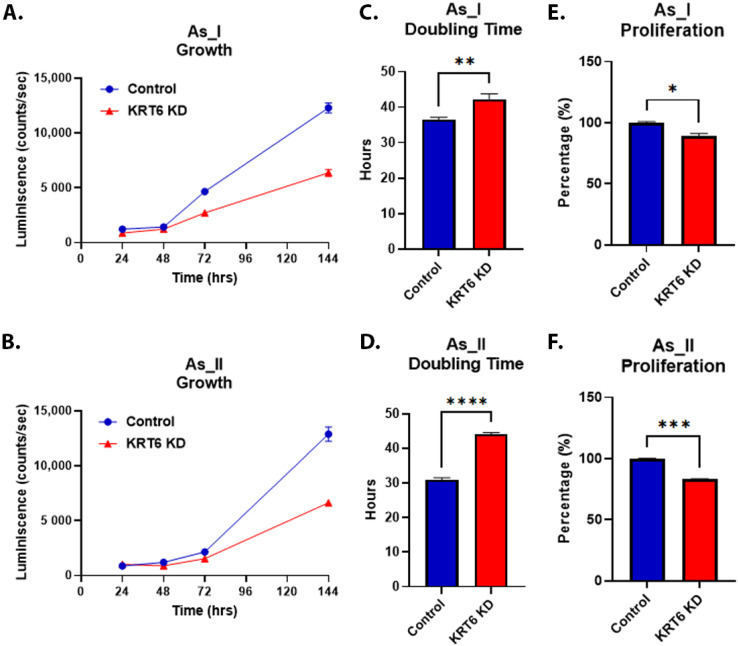
Growth of As^3+^-transformed UROtsa cells after KRT6 knockdown. (**A**,**B**) Luminescence intensity plotted over 24, 48, 72, and 144 h for control and KRT6 knockdown UROtsa As_I and As_II cells. A total of 1250 cells were initially seeded for growth and measured according to their luminescence on a 96-well plate. (**C**,**D**) Calculated doubling times obtained from the luminescence intensity curve. (**E**,**F**) Flow cytometric analysis of cell proliferation using Tag-it Violet labeling of cells 72 h after seeding 250,000 cells/well on a six-well plate. Luminescence growth represents *n* = 8 and the mean intensity ± SEM is plotted. For flow cytometry-based proliferation, duplicate measurements were performed for each condition (control or KRT6 knockdown) and the data are reported as the percentage of cells where the values represent mean ± SEM. A *t*-test was performed, and asterisks indicate significant differences from the control (* *p* < 0.05, ** *p* < 0.01, *** *p* < 0.001, **** *p* < 0.0001).

We utilized the luminescence assay to measure cell proliferation rates in the UROtsa As_I and As_II cells after KRT6 knockdown. Figure 6A,B show the luminescence intensity readings from 24 h, 48 h, 72 h, and 144 h and Figure 6C,D show the calculated doubling time of the As_I and As_II cells after KRT6 knockdown. The data show the KRT6 knockdown cells grow slower compared to their respective scramble control cells. To validate the luminescence growth assay results, we utilized a separate flow cytometry-based assay (Tag-It Violet™ Proliferation Assay) to measure cellular proliferation (Figure 6E,F). The results from this experiment confirm that the knockdown of KRT6 in UROtsa As_I and As_II cells slows their proliferation.

### 3.7. Colony Formation of As^3+^-Transformed UROtsa Cells After KRT6 Knockdown

**Figure 7:**
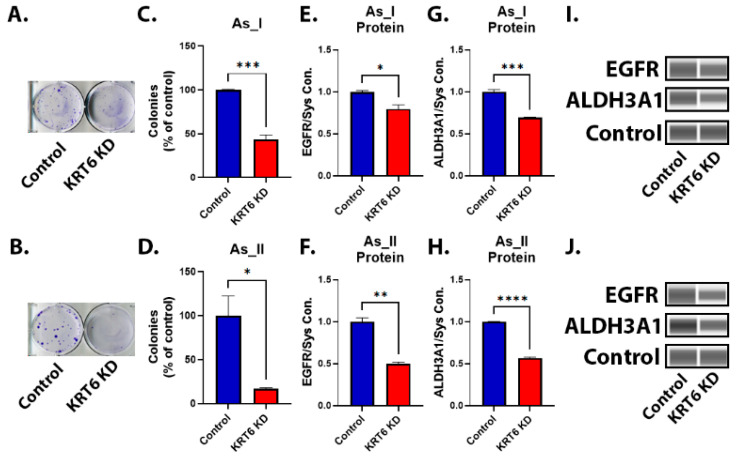
Colony formation of As^3+^-transformed UROtsa cells after KRT6 knockdown. (**A**,**B**) Image of crystal violet-stained colonies in the UROtsa As_I and As_II cells, respectively. A total of 200 cells/well were initially seeded per well in a six-well plate and were cultured for 14 days before staining the resulting colonies. There were triplicate seedings for control and KRT6 knockdown cells for each cell line (**C**,**D**). The quantification of colonies is mean of triplicate measurements expressed as percentage of control. (**E**,**F**) Quantification of EGFR protein from Western blot. (**G**,**H**) Quantification of ALDH3A1 protein from Western blot. (**I**,**J**) Western blot image of EGFR and ALDH3A1 expression after KRT6 knockdown in UROtsa As_I and As_II cells, respectively. Protein data are plotted as fold-change compared to the scramble control for each cell line. Protein expression was normalized to the Jess system control. The measurements were performed in duplicate for protein data. The reported values are mean ± SEM. A *t*-test was performed and asterisks indicate significant differences from the control (* *p* < 0.05, ** *p* < 0.01, *** *p* < 0.001, **** *p* < 0.0001).

In vitro colony formation measures the ability of a single cell to divide into a colony of 50 or more cells and is reflective of the stemness and reproductive viability of cells [42,43]. The results from the colony formation assay between scramble control and KRT6 knockdown cells show that the KRT6 knockdown cells form fewer colonies (Figure 7A–D) than the scramble control cells. Epidermal growth factor receptor (EGFR) and aldehyde dehydrogenase 3 family member A1 (ALDH3A1) play important roles in cancer stem cell metabolism [29,30]. Therefore, we measured the levels of these two proteins in the As_I and As_II cells after KRT6 knockdown. The results from Figure 7E–J show that both EGFR and the ALDH3A1 protein are lower in the KRT6 knockdown cells. Additionally, Figure S3I–L demonstrates that the mRNA transcript levels of these two genes are reduced in the KRT6 knockdown cells. Additionally, Figure S4I,J shows that in human bladder cancer cases, the expression of these two genes is also correlated to the expression of KRT6A.

### 3.8. Cisplatin Sensitivity of As^3+^-Transformed UROtsa Cells After KRT6 Knockdown

**Figure 8:**
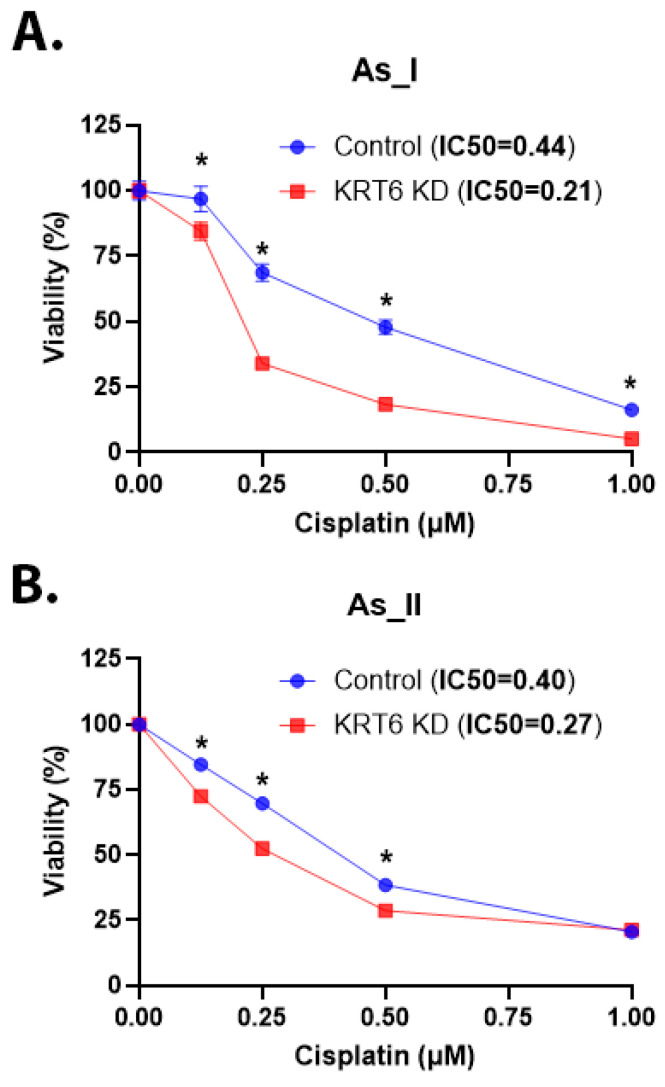
Cisplatin sensitivity of As^3+^-transformed UROtsa cells after KRT6 knockdown. (**A**,**B**) Viability (expressed in percentage) after 0.00 (control), 0.13, 0.25, 0.50, and 1 µM cisplatin treatment for 72 h in the UROtsa As_I and As_II cells, respectively. The IC50 values are reported in the top panel (next to sample names). Blue bars indicate control viability while red bars indicate viability in the KRT6 KD cells. 1250 cells were seeded/well in a 96-well plate and there were eight replicates for each dose. Twenty-four hours after seeding, the cells were dosed with either 0.00, 0.13, 0.25, 0.50, or 1 µM cisplatin treatment for 72 h. The reported values are mean ± SEM. A *t*-test was performed, and asterisks indicate significant differences from the control (* *p* < 0.05).

Cisplatin-based treatment followed by radical cystectomy is the standard treatment for MIUC and it has been reported that UC tumors with SD are less sensitive to cisplatin-based chemotherapy [12,44]. The UROtsa As_I and As_II cells are a model of the basal subtype of MIUC and, when injected into immunocompromised mice, the tumors derived from these cells form areas of SD that are enriched with KRT6 expression [17,20]. Therefore, we wanted to assess whether knocking down KRT6 expression in these cells would alter their sensitivity to cisplatin. We utilized the ATP-dependent luciferase assay to measure cell viability after cisplatin treatment for 72 h (Figure 8A,B). The results demonstrate lowered IC50 values for the KRT6 knockdown cells compared to the scramble control cells. The data confirm that the knockdown of KRT6 in As_I and As_II cells increases their sensitivity to cisplatin treatment. 

## 4. Discussion

Our work and the work of others have demonstrated that the expression of KRT6 is enriched within MIUC tumors [17,45]. The expression of KRT6 was found to be consistently higher in As^3+^-transformed UROtsa cells compared to parent (non-transformed) UROtsa cells. The tumor heterotransplants derived from As^3+^-transformed UROtsa cells highly express KRT6 and its expression correlates to areas of SD within those tumors [17,20]. Previously, we reported positive staining for KRT6 in high-grade human bladder cancer tumors but did not detect KRT6 protein expression in normal human urothelium [17]. In this current study, *KRT6A* transcript expression was shown to be expressed at very low levels in normal bladder tissue but was significantly elevated in bladder tumors, which confirms our previous results pertaining to KRT6A expression in the bladder. 

The basal subtype of MIUC is largely characterized by the expression of the basal keratins (KRT1, KRT5, KRT6, KRT14, KRT16). The luminal subtype is characterized by the expression of PPARG, FOXA1, and GATA3 transcription factors. In our study, we observed a decrease in basal keratins after KRT6 knockdown. To our knowledge, there are no studies that have knocked down KRT6 expression in urothelial cells and examined the expression of genes/proteins associated with the basal or luminal subtypes. However, in some studies, it has been shown that various small molecule treatments (retinoic acid, troglitazone) can reduce the markers of the basal subtype and induce the luminal markers [46,47,48]. Reversing a basal subtype of MIUC into a more luminal subtype is an interesting concept because this may correlate to better prognostic outcomes for patients. In the current study, we did not see any significant changes in the expression of these luminal markers (measured by gene expression) after knocking down KRT6 in the As_I and As_II cells. This suggests that the level of KRT6 in these cells does not regulate (directly or indirectly) the expression of the luminal transcription factors and that their levels are controlled by other mechanisms.

In the present study, we wanted to determine if KRT6 expression could regulate molecular or physiological processes in an in vitro model of the basal subtype of MIUC. We did not observe any morphological differences resulting from the reduction in KRT6 expression and evidence is lacking in terms of other reports of KRT6 knockdown in urothelial cancer cell lines. In fact, there are few studies that have reported knocking down KRT6 in any cell type. The studies that have knocked down KRT6, to our knowledge, have been performed in lung cancer and nasopharyngeal carcinoma cells and morphological changes were not demonstrated [49,50,51].

Several studies have analyzed the clinical significance of SD in UCs. These studies demonstrate that the presence of SD in UC tumors is associated with poor prognosis, decreased overall survival (OS), decreased recurrence-free survival (RFS), higher risk for metastasis, and being less responsive to cisplatin-based chemotherapy [12,13,52,53,54]. However, very little is known about the underlying mechanisms that regulate SD in UC and a better understanding of these mechanisms is important for designing more effective treatment options for patients with this disease. Warrick et al. demonstrated that SD is associated with the loss of expression of *FOXA1*, *GATA3*, and *PPARG* [41]. In this same study, they identified that FOXA1 regulates immune heterogeneity in bladder tumors with SD, an important finding pertaining to response to immune checkpoint inhibitors. They performed RNA-Seq on macrodissected regions of pure UC from SD tissue and identified several genes associated with SD; many of these were reduced after the knockdown of KRT6 in our study (*KRT5*, *KRT14*, *EGFR*, *KRT16*, *SPRR1A*, *SPRR2A*, and *SPRR3*). Desmocollin 2 (DSC2) is desmosomal protein that is a highly specific and sensitive immunohistochemical marker for distinguishing SD from pure UC; in our study, it was reduced after KRT6 knockdown [40]. Taken together, the results from our study demonstrated a reduction in several of these squamous-associated genes/proteins after the knockdown of KRT6 in As_I and As_II cells.

In lung cancer cell lines (H1299 and A549), it was demonstrated that KRT6 knockdown significantly inhibited proliferation and invasion [49]. In a separate study using lung cancer cell lines (A549 and PC-9), KRT6 knockdown reduced proliferation, migration, and colony formation [50]. In a human colon cancer cell line (DLD-1), KRT6A increased the proliferation, migration and invasion of the cells [55]. In our study, KRT6 knockdown in As_I and As_II cells resulted in decreased proliferation and a decrease in the colony-formation ability of the cells. These results are consistent with the data that have been published for other cell types. 

It has been reported that patients that have UC tumors with SD have a poor response to cisplatin-based treatments [12]. Studies are lacking that examine the effects of knocking down KRT6 in cisplatin-resistant UC cell lines. However, a study in human gastric carcinoma cells demonstrated that KRT6 was induced by chronic cisplatin treatment and that silencing KRT6 resulted in increased sensitivity to oxaliplatin, another platinum-based drug [56]. In the current study, we observed an increase in cisplatin sensitivity after we knocked down KRT6 expression in As_I and As_II cells.

## 5. Conclusions

In conclusion, this work was performed to gain a better understanding of the molecular mechanisms regulating SD in UC by silencing a key protein involved with this disease. The results demonstrate that inhibiting KRT6 expression in a basal subtype of MIUC can decrease the basal/squamous expression signature of the cells, decrease cell growth, and decrease colony formation. Furthermore, the data indicate that cisplatin sensitivity can be increased by decreasing KRT6 expression. This study lays the foundation for future in vivo studies examining the role of KRT6 knockdown in MIUC tumor pathology, specifically its effect on SD in the tumors and the effects it has on tumor growth and invasion, and assessments of whether the tumors are more responsive to cisplatin treatment. 

## Data Availability

The original contributions presented in this study are included in the article/Supplementary Material. Further inquiries can be directed to the corresponding author(s).

## Supplementary Materials

The following supporting information can be downloaded at: https://www.mdpi.com/article/10.3390/cells13211803/s1, Figure S1. Uncropped Western blots for Figure S1, Figure 1, Figure 2 and Figure 6. Figure S2. KRT6 protein expression after lentiviral knockdown in UROtsa As_I cells. Figure S3. Additional gene expression from control and KRT6 knockdown UROtsa As_I and As_II cells. Figure S4. Correlation of KRT6A gene expression in human bladder cancer. Table S1. List of primers used in the study. Table S2. Antibodies used for Western analysis.
