# Supplementary material for: Knockdown of Keratin 6 Within Arsenite-Transformed Human Urothelial Cells Decreases Basal/Squamous Expression, Inhibits Growth, and Increases Cisplatin Sensitivity
Source: Cells. 2024 Oct 31;13(21):1803. doi: 10.3390/cells13211803 (PMC11545824; doi:10.3390/cells13211803)
Supplement: Supplementary file 1 [file cells-13-01803-s001.zip › cells-3277168-supplementary.pdf]

Figure S1. Uncropped blots

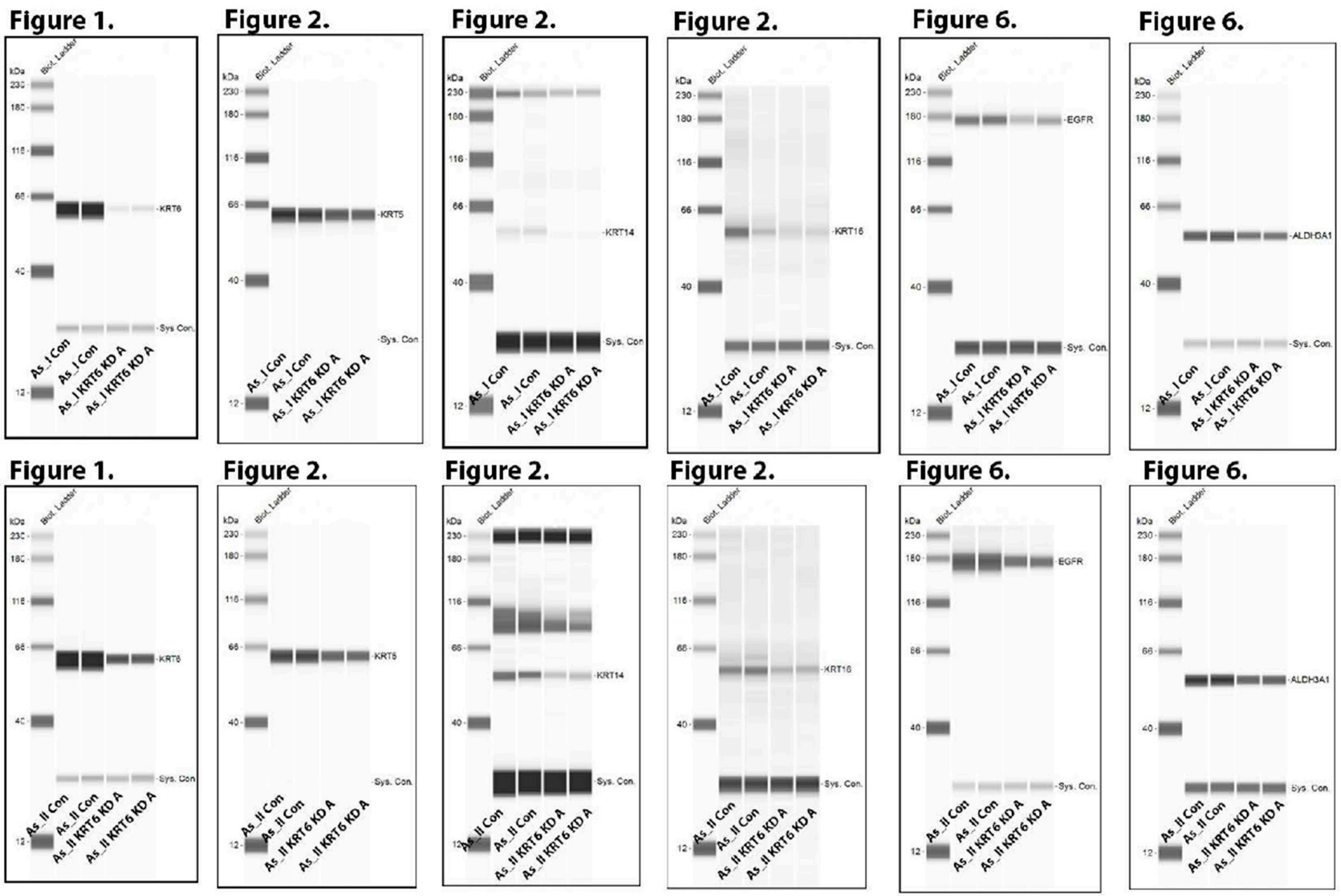

Figure S2. KRT6 protein expression after lentiviral knockdown in UROtsa As_I cells

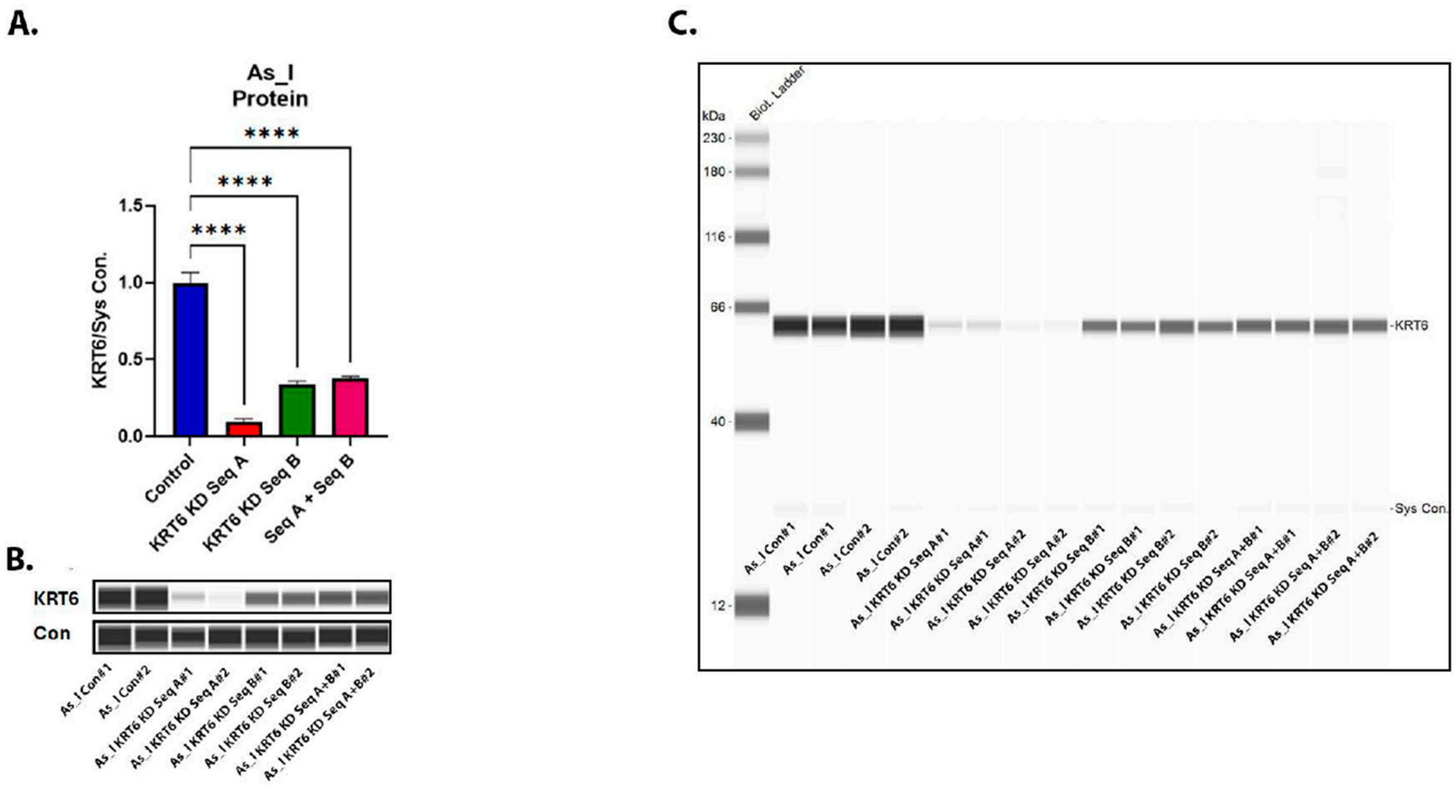

Figure S3. Gene expression

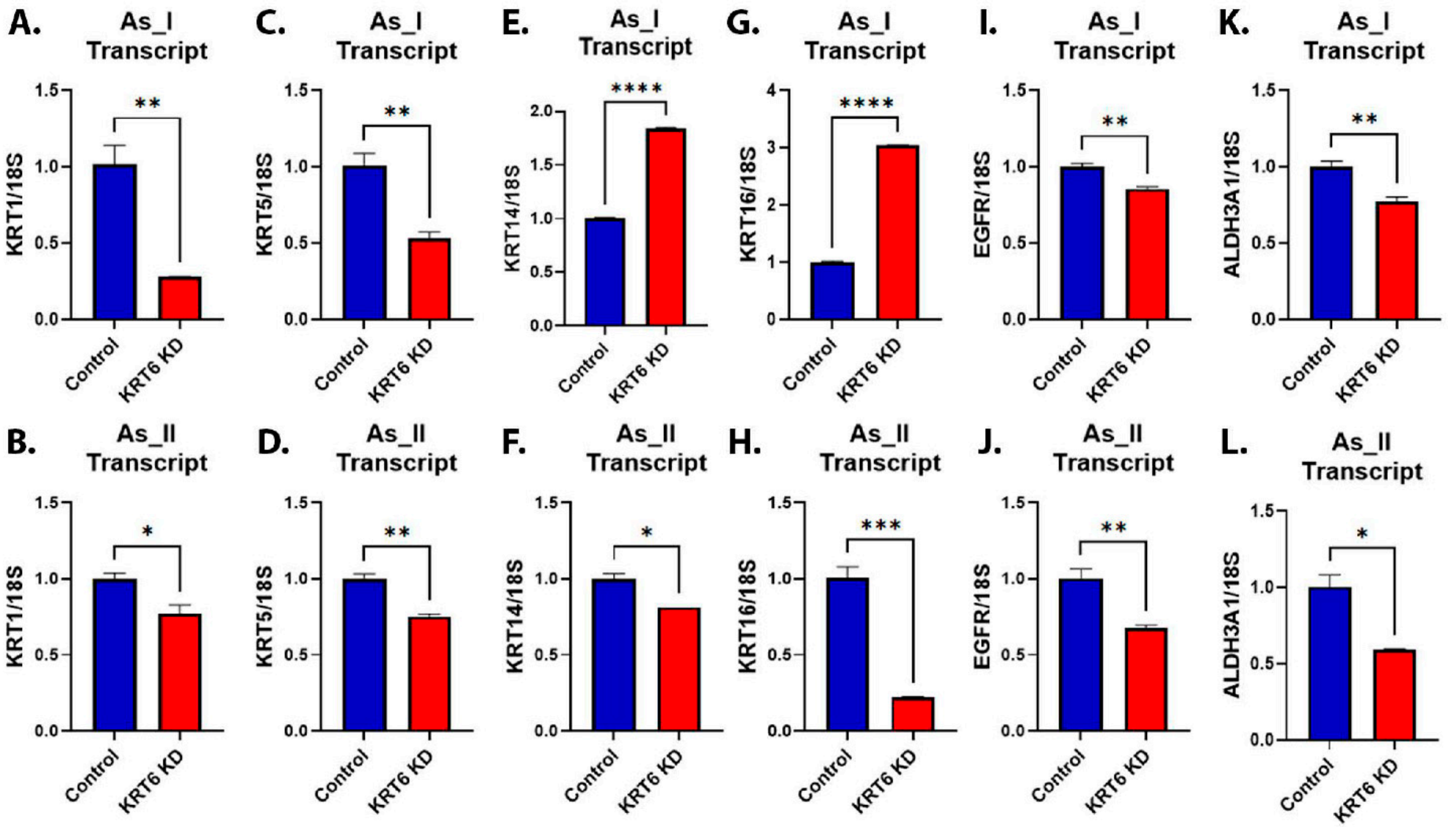

Figure S4. Correlation of KRT6 gene expression in human bladder cancer

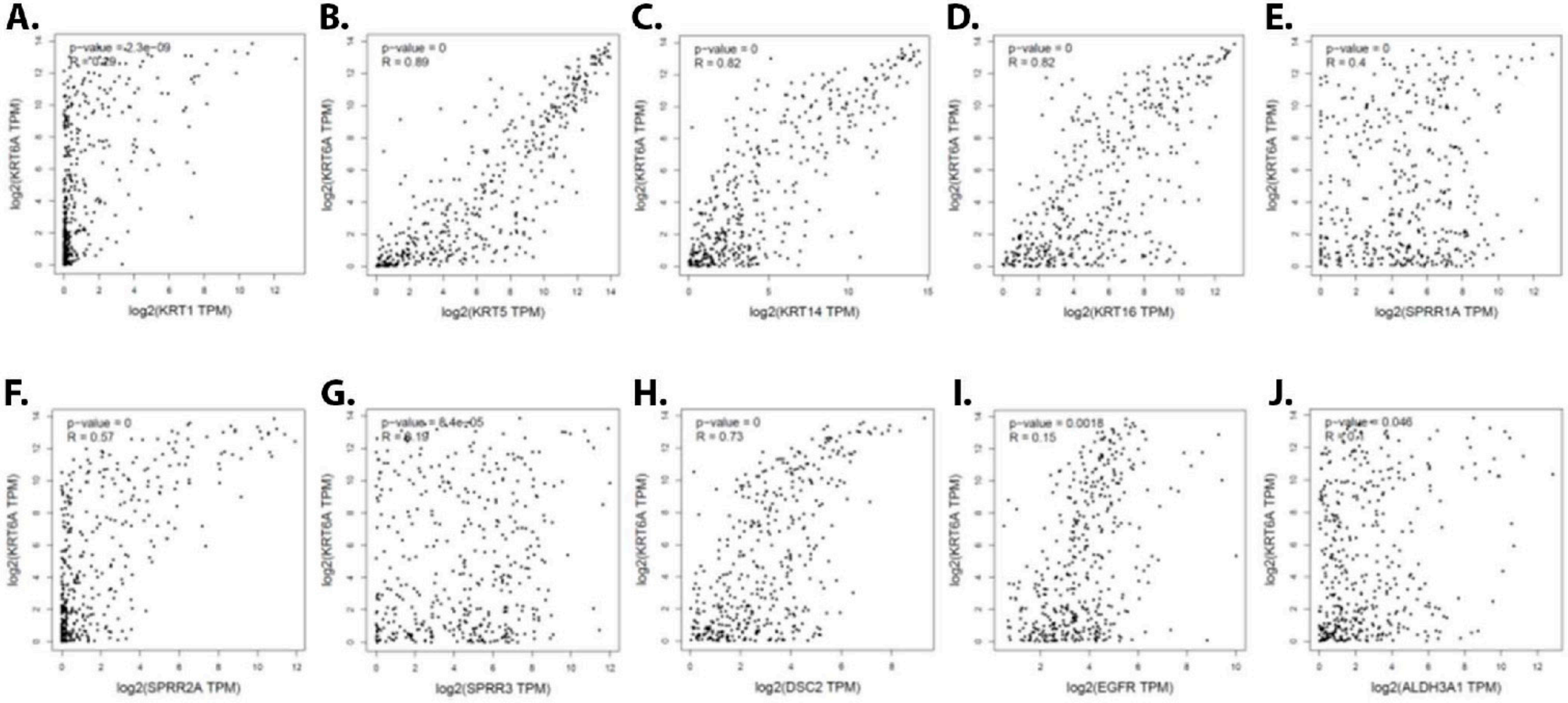

Table S1. List of primers used in the study

Genes	Product	Catalog No./unique Assay ID	Source
KRT1	PrimeTime qPCR primers	Hs.PT.58.24741966	Integrated DNA Technologies
KRT5	PrimeTime qPCR primers	Hs.PT.58.14446018	Integrated DNA Technologies
KRT6A	PrimeTime qPCR primers	Hs.PT.58.26132549.g	Integrated DNA Technologies
KRT14	PrimeTime qPCR primers	Hs.PT.58.4592110	Integrated DNA Technologies
KRT16	PrimeTime qPCR primers	Hs.PT.58.40837518.g	Integrated DNA Technologies
SPRR1A	PrimeTime qPCR primers	Hs.PT.58.39085888.g	Integrated DNA Technologies
SPRR2A	PrimeTime qPCR primers	Hs.PT.58.19341995	Integrated DNA Technologies
SPRR3	PrimeTime qPCR primers	Hs.PT.58.25686932.g	Integrated DNA Technologies
DSC2	PrimeTime qPCR primers	Hs.PT.58.931954	Integrated DNA Technologies
EGFR	PrimeTime qPCR primers	Hs.PT.58.15419889	Integrated DNA Technologies
ALDH3A1	PrimeTime qPCR primers	Hs.PT.56a.24823646.g	Integrated DNA Technologies

*18S rRNA primers were custom ordered from IDT- Upper: CGCCGCTAGAGGTGAAATTC
Lower: TTGGCAAATGCTTTCGCTC

Table S2. Antibodies used for Western analysis

Antigen	Source	Cat. No	Protein lysate conc. (µg/µL)	Dilution
KRT5	Invitrogen	PA5-29670	0.03	1:1600
KRT6A	Santa Cruz	sc-514520	0.03	1:1600
KRT14	Invitrogen	PA5-16722	0.06	1:200
KRT16	Abcam	ab76416	0.50	1:100
EGFR	Cell Signaling Technology	4267S	0.50	1:300
ALDH3A1	Novus	NBP2-47551	0.25	1:200

Supporting information legends

Figure S1. Uncropped Western blots for Figures S1, 1, 2, and 6. Displays all uncropped Western blot images used to generate figures 1, 2, and 6.

Figure S2. KRT6 protein expression after lentiviral knockdown in UROtsa As_I cells. Scramble (control) shRNA or different shRNA sequences targeting human KRT6 (sequence A, sequence B, or combination of sequence A + sequence B) were evaluated for ability to reduce KRT6 expression relative to the scramble control. (A) Quantification of KRT6 protein expression measured by Western blot after lentiviral knockdown of KRT6 in UROtsa As_I cells. (B) Representative Western blot image measuring KRT6 expression after lentiviral knockdown. (C) Uncropped Western blot image showing all biological (n=2) and technical replicates (n=2). Quantification data represented as mean ± SEM and plotted as fold-change from control. An ordinary one-way ANOVA was performed followed by a Dunnett's multiple comparisons test to demonstrate significance. Asterisks indicate significant differences from control values (****$p<0.0001$).

Figure S3. Additional gene expression from control and KRT6 knockdown UROtsa As_I and As_II cells. Gene expression from As_I cells is listed on top row and gene expression from As_II cells is listed on bottom row. The scramble control (blue bars) and KRT6 KD (red bars) expression are shown. (A,B) KRT1, (C,D) KRT5, (E,F) KRT14, (G,H) KRT16, (I,J) EGFR, (K,L) ALDH3A1. All data is plotted as fold-change compared to the scramble control. Gene expression was normalized to the 18S housekeeping gene. The gene measurements were performed in triplicates and the values reported are mean ± SEM. A t test was performed, and

asterisks indicate significant differences from the control (* $p < 0.05$, ** $p \leq 0.01$, *** $p \leq 0.001$, **** $p \leq 0.0001$).

Figure S4. Correlation of KRT6A gene expression in human bladder cancer. Human KRT6A gene expression was correlated (Pearson) to expression of (A) KRT1, (B) KRT5, (C) KRT14, (D) KRT16, (E) SPRR1A, (F) SPRR2A, (G) SPRR3, (H) DSC2, (I) EGFR, and (J) ALDH3A1 in human bladder cancer cases using GEPIA2 (Gene Expression Profiling Interactive Analysis, http://gepia2.cancer-pku.cn/#index). The web-based tool allows analysis based on the Cancer Genome Atlas (TCGA) and Genotype-Tissue Expression (GTEX) data.

Table S1. List of primers used in the study

Table S2. Antibodies used for Western analysis
